# Giant Left Main Coronary Artery Aneurysm Presenting as Multiple Implantable Cardioverter Defibrillator Shocks

**DOI:** 10.7759/cureus.7653

**Published:** 2020-04-12

**Authors:** Yury Malyshev, Asma Syed, Ricardo Castillo, Rumman A Syed, Sonu Sahni

**Affiliations:** 1 Cardiology, Maimonides Medical Center, Brooklyn, USA; 2 Cardiology, Brookdale University Hospital Medical Center, Brooklyn, USA; 3 Internal Medicine, Brookdale University Hospital Medical Center, Brooklyn, USA; 4 Research Medicine, New York Institute of Technology College of Osteopathic Medicine, New York, USA; 5 Primary Care, Touro College of Osteopathic Medicine, New York, USA

**Keywords:** coronary artery aneurysm, defibrillator shock, ventricular tachycardia, left main coronary artery aneurysm, left main aneurysm

## Abstract

Giant aneurysms of the left main coronary artery are one of the rarest findings in cardiology, encountered in less than 0.02% of patients. The presentation is usually the same as coronary artery disease since most coronary aneurysms in the western world are associated with atherosclerosis. Here we report the first case of giant aneurysm of the left main coronary artery presenting as ventricular tachycardia with multiple shocks of the defibrillator in a 57-year-old man with heart failure. We also review the etiology, pathology, and management of coronary aneurysms.

## Introduction

Coronary artery aneurysms (CAAs) are very rare clinical entities; among them giant left main coronary artery aneurysms (LMCAAs) are exceedingly rare, encountered in less than 0.02% of patients [1]. Etiology of CAA varies depending on age, comorbidities and even geographical area. The etiology usually determines presentation and management. Herein we report a unique case of giant LMCAA in a 57-year-old man with heart failure with reduced ejection fraction (HFrEF), who presented to our emergency department with chest pain after his implantable cardioverter defibrillator (ICD) fired 12 times. Urgent diagnostic catheterization showed giant LMCAA without signs of coronary artery disease (CAD). The patient was started on dual antiplatelet therapy. He remained asymptomatic for more than a year. We also review current literature on various diagnostic modalities and different management approaches of CAAs. 

## Case presentation

**Figure 1:**
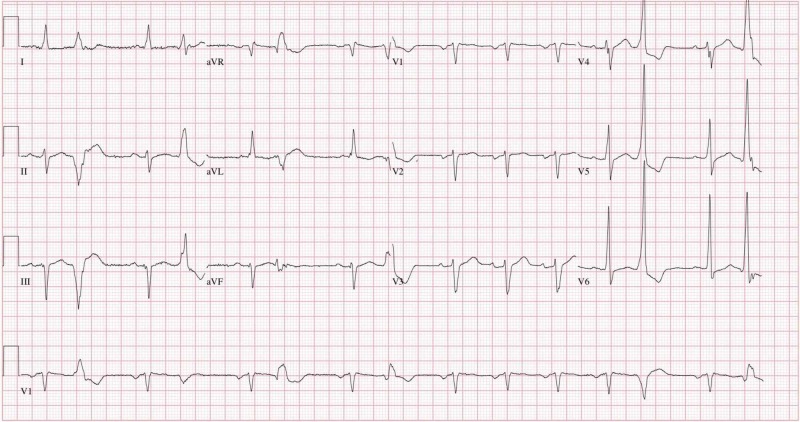
EKG showing sinus rhythm with premature ventricular contractions and left anterior fascicular block

A 57-year-old man with hypertension, diabetes, obesity, and HFrEF presented with chest pain, palpitations, and syncope the day before. His ICD fired 12 times. His vitals were unremarkable. Physical examination was significant for irregular pulse. EKG showed normal sinus rhythm with frequent premature ventricular complexes and left anterior fascicular block (Figure 1).

Chest x-ray was clear. Blood work showed elevated cardiac enzymes and electrolyte abnormality, and drug screen was positive for cannabinoid (Table 1).

**Table 1:** Significant lab results CPK, creatine phosphokinase; pBNP, pro-brain natriuretic peptide; ANA, antinuclear antibody; anti-dsDNA, anti-double-stranded DNA antibody; pANCA, perinuclear antineutrophil cytoplasmic antibodies

Test	Result
Troponin (ng/mL)	2.14
CPK (U/L)	537
pBNP (pg/mL)	1860
Potassium (mEq/L)	2.8
ANA	Negative
Anti-dsDNA antibody (IU/mL)	1
pANCA (AU/mL)	<1.0
C3 complement (mg/dL)	202
C4 complement (mg/dL)	40
Drug screen	Cannabinoids

ICD interrogation showed that two shocks were administered for ventricular tachycardia and 10 shocks were inappropriate due to electromagnetic interference on the lead. Echocardiogram revealed ejection fraction of 10%-15% with diffuse hypokinesis (Video 1).

**Video 1:** Echocardiogram showing severely reduced left ventricular systolic function, ejection fraction of 10%-15%, and diffuse hypokinesis

**Figure 2:**
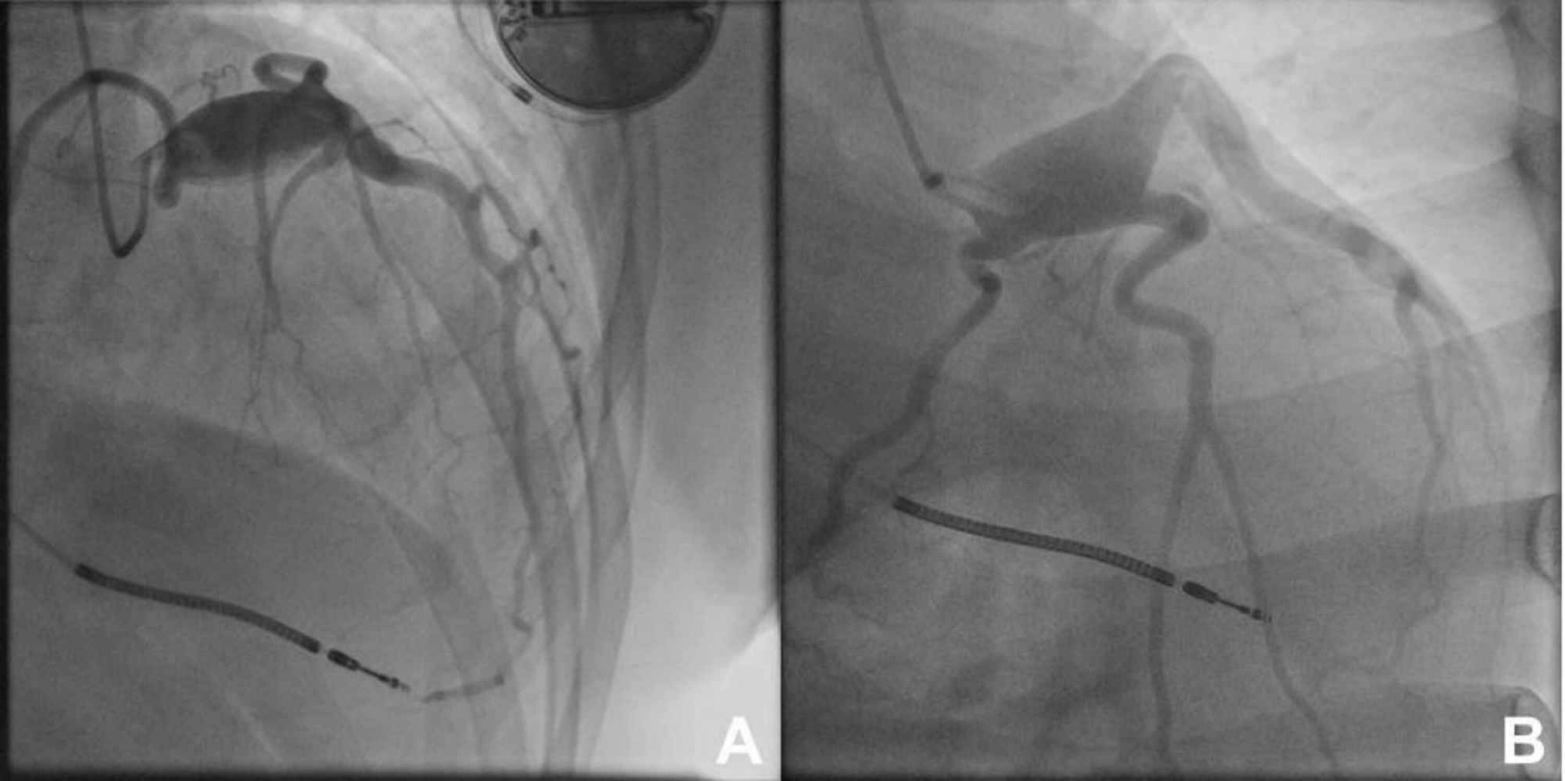
Giant aneurysm of the left main coronary aneurysm A: right anterior oblique cranial view; B: right anterior oblique caudal view

Urgent cardiac catheterization showed no evidence of occlusive CAD. There was however a large saccular LMCAA involving the ostium of the left anterior descending (LAD), left circumflex (LCX), and ramus intermedius arteries. The size of the aneurysm was measured to be 37.4 mm x 20 mm (Figure 2, Video 2). Autoimmune workup was negative (Table 1).

**Video 2:** Angiogram showing giant aneurysm of the left main coronary artery

**Figure 3:**
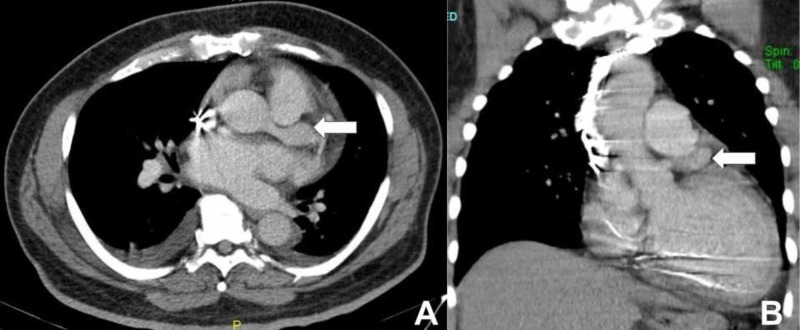
CT chest showing left main coronary artery aneurysm (white arrow) A: transverse plane; B: coronal plane

The patient was started on dual antiplatelet therapy with aspirin and clopidogrel. CT surgery evaluated the patient, but did not recommend intervention. The patient had successful implantation of a cardioverter defibrillator during the same admission. He was seen in ED one year later with suspicion for pulmonary embolism. CT chest angiogram showed LMCAA to be 1.5 cm in diameter (Figure 3).

Two months after the ED visit, he was seen in the clinic symptom free and ICD was functioning well. 

## Discussion

**Figure 4:**
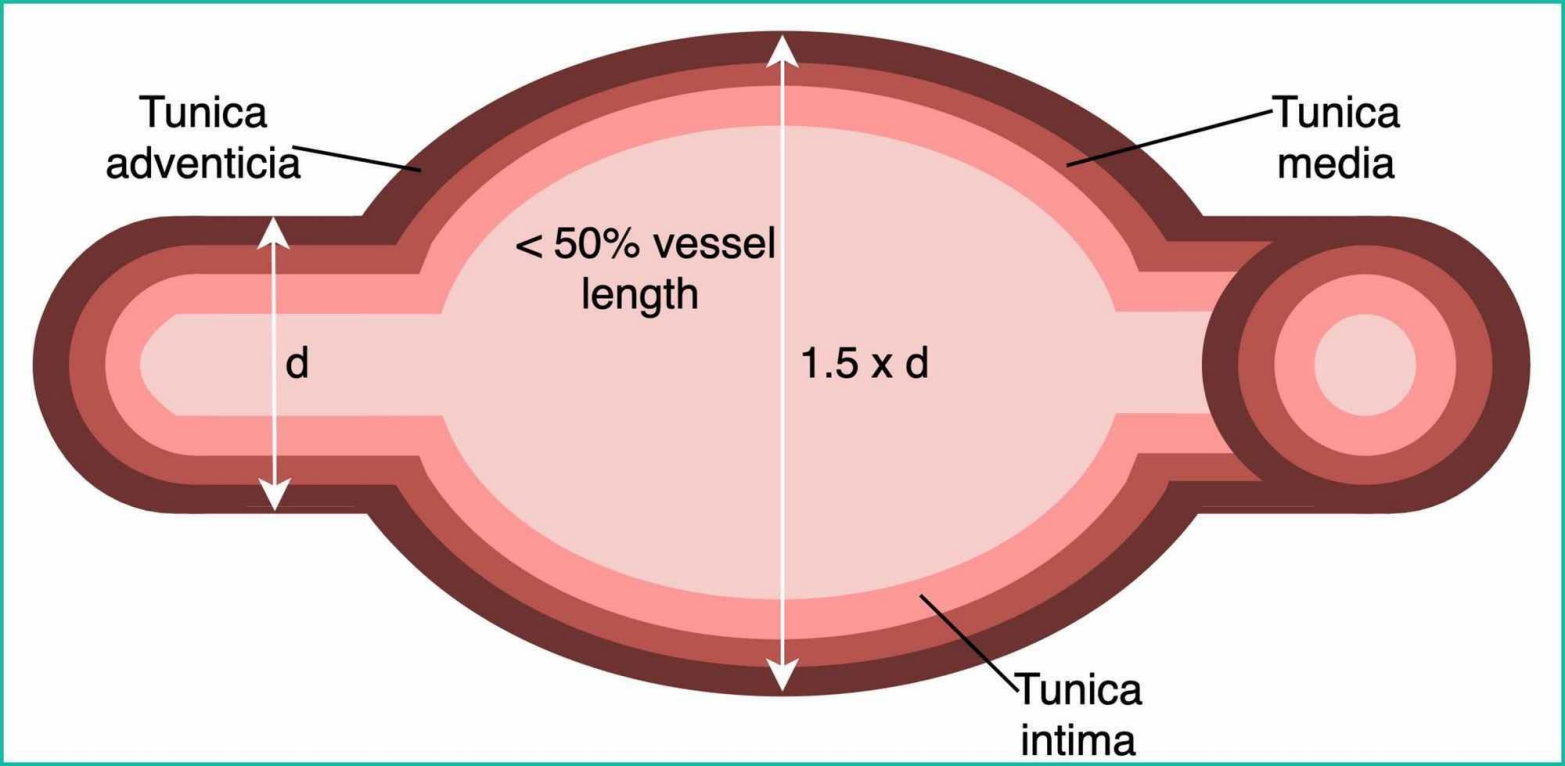
Schematic representation of a true coronary aneurysm d, diameter

CAA is a segment of the artery with width greater than length and diameter greater than diameter of a normal adjacent segment or 1.5 times larger than the largest coronary vessel (Figure 4) [2,3].

CAAs are classified as follows. Wall composition: true aneurysms have all three vessel wall layers; pseudoaneurysms lose one or two. Shape: saccular CAAs transverse diameter is greater than the longitudinal diameter. They are often seen distal to stenosis and are more prone to thrombosis or rupture. Fusiform aneurysms involve the whole vessel circumference, have greater longitudinal measurement, and have no relationship to stenosis. Size: small (diameter <5 mm), medium (5-8 mm), and giant (>8 mm) [3].

**Figure 5:**
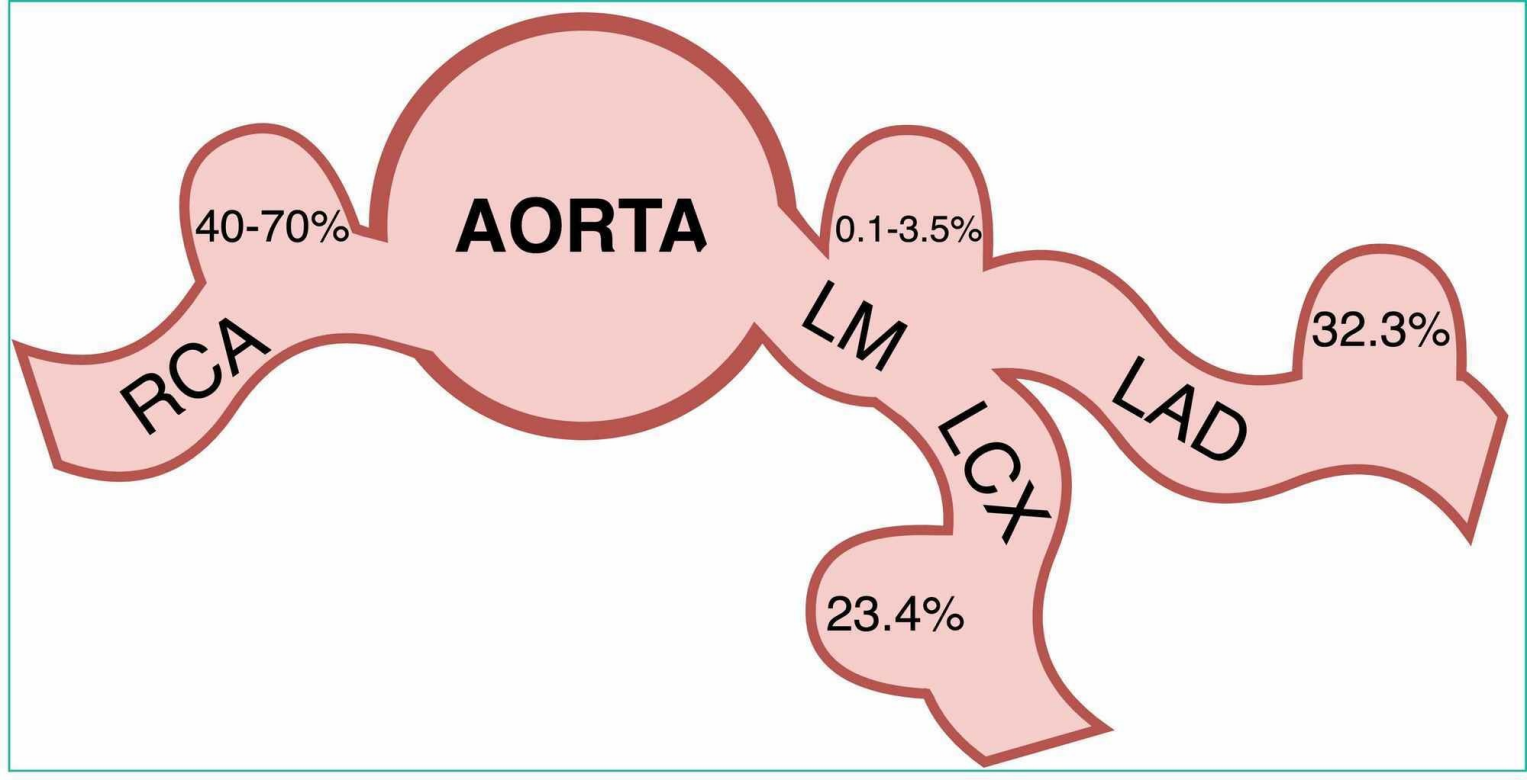
Distribution and fréquence of coronary artery aneurysms LM, left main coronary artery; LAD, left anterior descending artery; LCX, left circumflex artery; RCA, right coronary artery

Incidence varies from 0.3% to 5.3% (mean of 1.65%). Men have more CAAs than women: 2.2% vs. 0.5%. Most frequent locations are right coronary (40%-70%), LCX (23.4%), and LAD (32.3%) arteries. Left main coronary artery is affected significantly less (0.1%-3.5%) (Figure 5) [4,5]. Prevalence of giant CAA in a general population is only 0.02% [1]. 

The etiology of CAA varies depending on age and geographical area. Atherosclerosis is responsible for half of CAAs in the West, followed by congenital (17%) and infectious (10%) aneurysms. Kawasaki disease is the dominant cause of CAA in Japan [3]. Inflammatory disorders and connective tissue diseases are usually associated with ectasias and are more frequent in younger patients [3]. Iatrogenic causes include trauma from balloon inflation pressure, intervention in acute myocardial infarction, use of nonsteroidal anti-inflammatory drugs, steroids, and colchicine, which can cause improper healing. Cocaine causes severe hypertension and vasoconstriction, thus damaging the endothelium and promoting CAA formation [6].

CAAs in CAD are thought to be caused by turbulent blood flow damaging the wall [3]. However, there must be other factors because most patients with CAD do not develop CAA.

Usually patients are asymptomatic and most CAAs are found incidentally. Presentation depends on the etiology and/or complications. Complications of CAA include embolization, rupture, fistula formation, tamponade, hemopericardium, dissection, vasospasm, and vessel compression [2,3].

Coronary angiography remains the best method to identify CAA [2]. It provides information about location, size, and shape of CAA, but it only sees the vessel lumen. Thus, the true size of CAA could be underestimated, or CAA can be missed if thrombus occludes it [4]. Intravascular ultrasound corrects these limitations, providing transmural images and information about wall structure and luminal composition [4,7]. CT coronary angiography provides fast information about CAA’s location, shape, size, and wall composition, but no treatment option. CT angiography is useful in following patients with known CAAs [4].

Management of CAA depends on presentation, etiology, size, location, associated infection, and extent of atherosclerosis [4]. In adults with CAD, medical reduction of cardiovascular risk factors should be started. Long-term antiplatelets and potentially anticoagulation should be started since thrombosis and/or embolism are of concern [8]. Percutaneous intervention with stent placement can be done in aneurysm with diameter up to 10 mm [9]. Surgery is indicated in patients, who are not candidates for percutaneous intervention, obstructive CAD, and large saccular aneurysms at risk for rupture [4,10].

## Conclusions

The first case of CAA was published in 1812. It was found post-mortem after sudden death. Since then our understanding of the pathology, etiology, and progression of CAAs has improved. Today we can find these potentially deadly aneurysms during routine angiogram, not post-mortem. However, their management is still challenging and has to be tailored specifically to each patient. More research is needed to identify patients, who are at risk to diagnose CAA earlier, manage it better, and prevent complications. 
